# Metabolic Changes Induced by Silver Ions in *Carlina acaulis*

**DOI:** 10.3390/plants8110517

**Published:** 2019-11-17

**Authors:** Sławomir Dresler, Barbara Hawrylak-Nowak, Maciej Strzemski, Magdalena Wójciak-Kosior, Ireneusz Sowa, Agnieszka Hanaka, Iwona Gołoś, Agnieszka Skalska-Kamińska, Małgorzata Cieślak, Jozef Kováčik

**Affiliations:** 1 Department of Plant Physiology and Biophysics, Institute of Biological Science, Maria Curie-Skłodowska University, 20-033 Lublin, Poland; agnieszka.hanaka@poczta.umcs.lublin.pl (A.H.); fantan8@wp.pl (I.G.); 2 Department of Botany and Plant Physiology, Faculty of Environmental Biology, University of Life Sciences in Lublin, Akademicka 15, 20-950 Lublin, Poland; 3 Department of Analytical Chemistry, Medical University of Lublin, 20-093 Lublin, Poland; maciej.strzemski@poczta.onet.pl (M.S.); kosiorma@wp.pl (M.W.-K.); i.sowa@umlub.pl (I.S.); agnieszka.skalska-kaminska@umlub.pl (A.S.-K.); 4 Łukasiewicz—Textile Research Institute, Scientific Department of Unconventional Technologies and Textiles, Brzezińska 5/15, 92-103 Łódź, Poland; cieslakm@iw.lodz.pl; 5 Department of Biology, University of Trnava, Priemyselná 4, 918 43 Trnava, Slovak Republic; jozkovacik@yahoo.com

**Keywords:** heavy metals, thiols, phenolic metabolites, organic acids

## Abstract

Silver is one of the most toxic heavy metals for plants, inducing various toxic symptoms and metabolic changes. Here, the impact of Ag(I) on *Carlina acaulis* physiology and selected metabolites was studied using two Ag concentrations (1 or 10 µM) after 14 days of exposure. The higher concentration of Ag(I) evoked reduction of growth, while 1 µM Ag had a growth-promoting effect on root biomass. The translocation factor (<0.04) showed that Ag was mainly retained in the roots. The 1 µM Ag concentration increased the level of low-molecular-weight organic acids (LMWOAs), while 10 µM Ag depleted these compounds in the roots. The increased concentration of Ag(I) elevated the accumulation of phytochelatins (PCs) in the roots and reduced glutathione (GSH) in the shoots (but not in the roots). At 1 µM, Ag(I) elevated the level of phenolic and triterpene acids, while the 10 µM Ag treatment increased the carlina oxide content in the roots. The obtained results indicate an alteration of metabolic pathways of *C. acaulis* to cope with different levels of Ag(I) stress. Our data imply that the intracellular binding of Ag(I) and nonenzymatic antioxidants contribute to the protection against low concentrations of Ag ions.

## 1. Introduction

The exposure of plants to toxic levels of heavy metals (HMs) induces many changes in their metabolism [1,2,3]. In response to metal stress, plants activate several HM tolerance mechanisms, including enzymatic and nonenzymatic antioxidant systems or HM-binding compounds such as phytochelatins or organic acids [4]. Moreover, HM excess has a typically significant impact on the synthesis and accumulation of secondary metabolites (SMs) [5,6]. The role of SMs in HM detoxification may be related to the presence of hydroxyl groups in phenolic compounds, while thiols or ascorbate are generally protective compounds in plants [7,8,9].

Among HMs, Ag ions are considered as one of the most toxic agents for plants [9]. Their presence in the environment leads to several negative and toxic symptoms, including growth reduction, disruption of macro- and micronutrient uptake and homeostasis, or depletion of photosynthetic pigments [9,10]. As the environmental pollution with this metal has increased over the past few decades owing to human activities [11], the influence of Ag on living organisms has received increasing attention [12,13].

*Carlina acaulis* L. from the Asteraceae family is a monocarpic perennial herb. Xerothermic and calcareous grasslands are natural habitats of these plants [14]. However, *Carlina* genus plants are also found in HM-contaminated areas, for example, waste deposits from metal mining and smelting [15]. As a pseudometallophyte, *C. acaulis* can be an attractive object to study the HM tolerance and detoxification mechanisms. Various biochemical pathways leading to the production of a spectrum of metabolites such as triterpenes (oleanolic acid and ursolic acid), essential oil compounds (carlina oxide), or phenolic acids (chlorogenic acid and 3,5-dicaffeoylquinic acid) make this species ideal for the research of SM–HM interactions.

The aim of this study was to investigate the metabolic response of *C. acaulis* exposed to two concentrations of Ag(I) (1 or 10 µM). The objectives of the study included (i) estimation of *C. acaulis* tolerance to Ag(I) stress, (ii) assessment of Ag accumulation and translocation as well as the content of mineral nutrients, (iii) evaluation of nonenzymatic antioxidants and ligands, and (iv) assessment of the impact of Ag(I) on the production of secondary metabolites.

## 2. Results

### 2.1. Impact of Ag(I) on Plant Growth and H_2_O_2_ Accumulation

**Figure 1:**
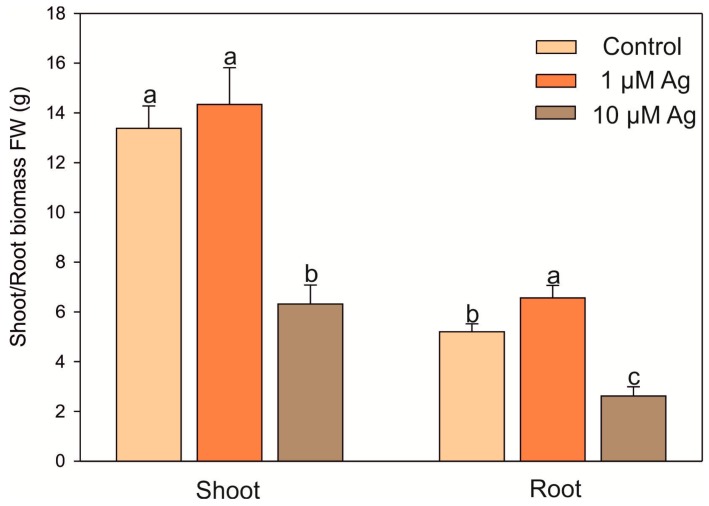
Effect of Ag(I) ions on the fresh weight of *Carlina acaulis* shoots and roots after 14 days of exposure. Data are mean ± SE (n = 25); values followed by the same letter are not significantly different (*p* < 0.05, Tukey’s test).

**Figure 2:**
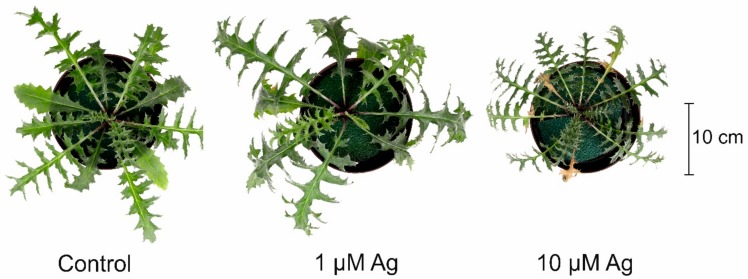
Phenotype of *C. acaulis* plants cultivated for 14 days at various Ag(I) concentrations.

**Figure 3:**
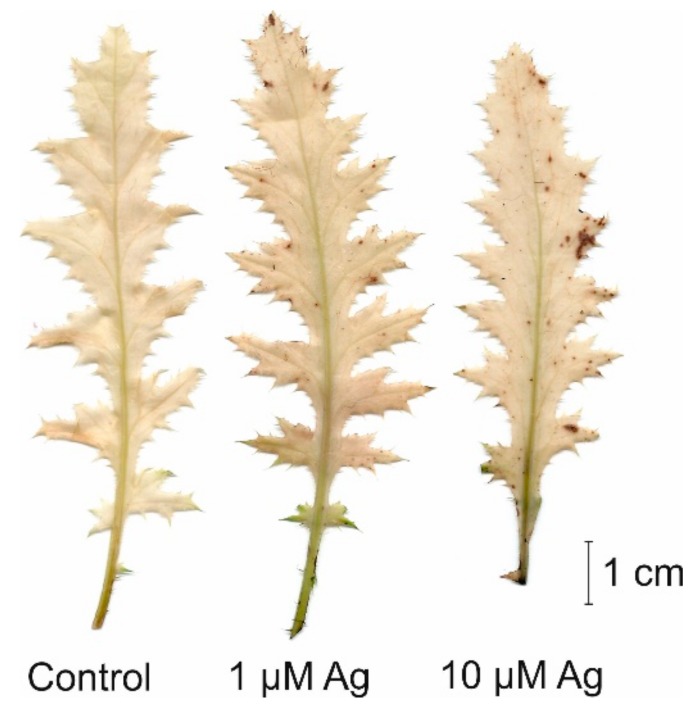
Histochemical detection of H_2_O_2_ in the leaves of *C. acaulis* growing for 14 days in the control conditions and at 1 or 10 µM Ag.

Both the shoot and root biomass of *C. acaulis* was variously affected by the two concentrations of Ag(I) applied to the cultivation solution (Figure 1). It was found that, contrary to 1 µM Ag, 10 µM Ag caused a reduction of shoot fresh weight (FW) by more than 52% in comparison with the control. Moreover, the higher Ag(I) concentration resulted in visible necrotic symptoms on the leaves, while the presence of 1 µM Ag did not negatively affect the phenotype (Figure 2). Similar to shoots, the higher concentration of Ag(I) reduced root biomass (50% of the control). Interestingly, plants exposed to 1 µM Ag showed significantly higher root biomass (126% of the control). Although there was no negative effect of 1 µM Ag on the shoot FW, this treatment slightly increased the H_2_O_2_ accumulation in the leaves (Figure 3). Contrary to the control leaves, both Ag(I) concentrations induced appearance of brown spots after 3′,3-diaminobenzidine (DAB) staining, which indicated induction of H_2_O_2_ accumulation.

### 2.2. Bioconcentrations of Ag and Selected Mineral Nutrients

**Figure 4:**
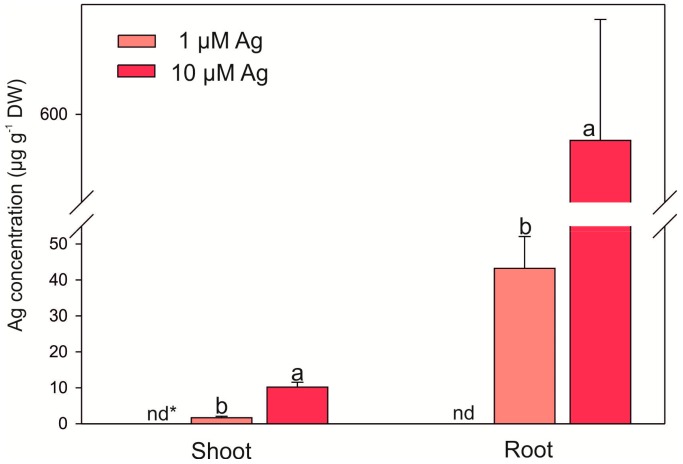
Ag concentrations in the roots and shoots of *C. acaulis* grown under two Ag(I) treatments for 14 days. Data are mean ± SE (n = 4); values followed by a different letter are significantly different within a particular organ (*p* < 0.05, Tukey’s test); * nd—not detected.

The accumulation of Ag in *C. acaulis* was significantly affected by its concentration in the nutrient medium (Figure 4). In the 10 µM treatment, the concentration of Ag in the shoots and roots significantly increased, reaching 6.1- and 13.6-fold higher levels in comparison with 1 µM Ag. It was also found that Ag was poorly transferred to the aboveground parts of the plants. The values of the Ag translocation factor (TF) calculated as the ratio of the Ag content in the shoots and roots (TF = 0.04 for 1 µM Ag; TF = 0.02 for 10 µM Ag) indicate that most Ag(I) ions taken up by roots are retained in these organs.

The concentrations of both K and P in the roots and Ca in the shoots decreased at 10 µM Ag (Table 1). In turn, the higher concentration of Ag caused an increase in the level of Ca in the roots, while plants exposed to 1 µM Ag contained more Ca in their shoots. Moreover, it was observed that the lower concentration of Ag ions significantly decreased the Cu level in the shoots. However, the accumulation of K and P in the shoots, Mg and Zn in both the shoots and roots, and Cu in the roots did not differ significantly between the treatments.

### 2.3. Changes in Low-Molecular-Weight Organic Acids (LMWOAs), Thiols, and Ascorbic Acid (AsA) Accumulation under Ag(I) Exposure

**Figure 5:**
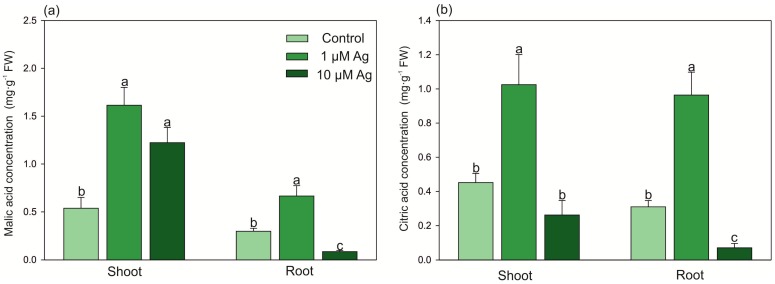
Effect of Ag(I) on (**a**) malic acid and (**b**) citric acid concentrations in the shoots and roots of *C. acaulis* after 14 days of exposure. Data are mean ± SE (n = 5); values for individual organs followed by the same letter are not significantly different (*p* < 0.05, Tukey’s test).

In the 1 µM Ag treatment, the contents of LMWOAs (malic and citric acids) in the shoots and roots were elevated (Figure 5): malate and citrate represented 304% and 227% of the control value in the shoots and 230% and 320% of the control value in the roots, respectively. Similarly, the higher concentration of Ag(I) also elevated the malic acid amount in the shoots. On the contrary, plants grown at 10 µM Ag showed a decrease in the malate and citrate content by 70% and 77%, respectively, compared with the control.

**Figure 6:**
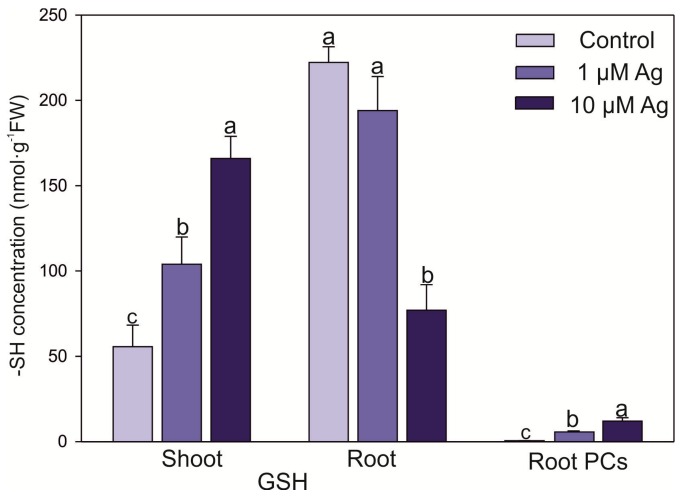
Effect of Ag(I) on reduced glutathione (GSH) concentrations in the shoots and roots and the total content of phytochelatins (PCs) in the roots of *C. acaulis* after 14 days of exposure. Data are mean ± SE (n = 5); values for individual organs followed by the same letter are not significantly different (*p* < 0.05, Tukey’s test).

The exposure to Ag increased the level of reduced glutathione (GSH) in the shoots (Figure 6). The highest concentration of this thiol-peptide (approx. 3-fold higher than in the control) was found at 10 µM Ag. In turn, the higher Ag(I) concentration significantly depleted the level of GSH in the roots. The accumulation of phytochelatins (PCs) in the shoots was below the detection limit, while their accumulation in the roots was dependent on the Ag(I) dose. It was found that Ag ions stimulated the PC accumulation from 0.05 to 7.70 and 21.42 nmol -SH g^−1^ FW in the control, 1 µM Ag, and 10 µM Ag treatments, respectively.

**Figure 7:**
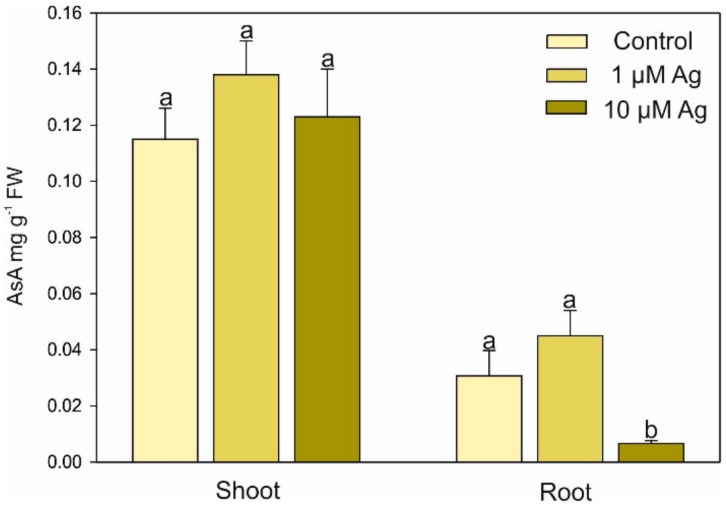
Effect of Ag(I) on ascorbic acid (AsA) concentrations in the shoots and roots of *C*. *acalulis* after 14 days of exposure. Data are mean ± SE (n = 5); values for individual organs followed by the same letter are not significantly different (*p* < 0.05, Tukey’s test).

The level of AsA in the shoots did not differ between the Ag(I) treatments; however, significant depletion of the AsA content in the roots was found at 10 µM Ag (Figure 7).

### 2.4. Changes in the Level of Selected Secondary Metabolites under Ag(I) Exposure

**Figure 8:**
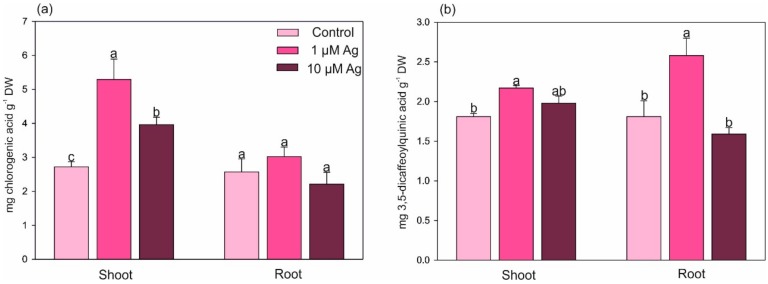
Effect of Ag(I) on the concentrations of (**a**) chlorogenic acid and (**b)** 3,5-dicaffeoylquinic acid in the shoots and roots of *C. acalulis* after 14 days of exposure. Data are mean ± SE (n = 5); values for individual organs followed by the same letter(s) are not significantly different (*p* < 0.05, Tukey’s test).

Two major phenolic acids, namely, chlorogenic acid (Figure 8a) and 3,5-dicaffeoylquinic acid (Figure 8b), were quantified in the shoots and roots of *C. acaulis.* Contrary to the roots, where no changes were found, the level of chlorogenic acid was significantly elevated in the shoots in response to the Ag(I) exposure and its concentrations increased by 2.9- and 1.5-fold at 1 and 10 µM Ag compared with the control. Only the lower Ag(I) dose had a significant positive effect on the concentration of 3,5-dicaffeoylquinic acid both in the shoots and roots.

**Figure 9:**
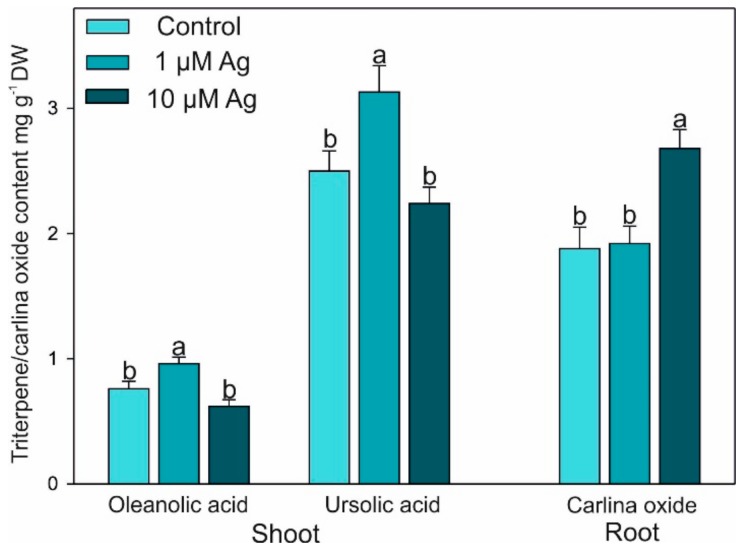
Effect of Ag(I) on the concentration of triterpene acids in the shoots and carlina oxide level in the roots of *C. acalulis* after 14 days of exposure. Data are mean ± SE (n = 5); values for individual organs followed by the same letter are not significantly different (*p* < 0.05, Tukey’s test).

The triterpene (oleanolic and ursolic) acids were detected in the shoots only, while carlina oxide was present in the root tissues only (Figure 9). The content of oleanolic and ursolic acids significantly increased under 1 µM Ag only. On the contrary, only the higher concentration of Ag(I) elevated the accumulation of carlina oxide in the roots.

## 3. Discussion

Reduction of growth is one of the most visible toxicity symptoms of HM excess in plants, and the negative impact of toxic concentrations of Cd, Pb, Cu, Zn, Ni, or Mn on plant biomass has been reported repeatedly [1,5]. Since Ag ions are highly toxic, exposure of plants to Ag evokes many negative effects, including reduction of biomass or decreased chlorophyll accumulation [16]. In agreement with these findings, 10 µM Ag(I) depleted the shoot and root biomass and produced visible necrotic symptoms on the leaves. On the contrary, the lower Ag(I) concentration slightly but significantly stimulated root biomass, indicating the hormetic effect of this dose. Similarly, the stimulatory effect of low Ag doses on plant growth has been previously reported [10,17,18,19,20]. Yang et al. [17] indicated that the exposure of rice plants to 50 µg L^−1^ Ag increased the root biomass by 52% and the root length by 72%. A beneficial effect of sublethal concentrations of Ag (<0.02 mg L^−1^) was also observed in *Arabidopsis thaliana* [18]. Also, our preliminary study showed that long-time exposure of *C. acaulis* to 0.1 µM Cd positively influenced the shoot and root biomass (unpublished data). The phytostimulative impact of a low concentration of HMs, the so-called hormetic effect, is probably an adaptive process [17,21]. The impact of Ag on ethylene biosynthesis (antagonist of its biosynthesis) may also play a role in the observed growth changes [22]. Moreover, it cannot be excluded that the hormetic effect was the result of the positive impact of the low Ag(I) concentration on the uptake and contents of some nutrients, for example, Ca in the leaves.

In this study, Ag was accumulated mostly in the roots. This finding is in agreement with previous reports, which showed that the translocation of this element to the aboveground parts of plants is very low in various species [9,18,23]. As reported by Wang et al. [18], Ag ions are quickly taken up by roots from the nutrient solution. They found distribution of Ag in hydroponically cultured *A. thaliana* mainly in roots (68%), followed by 30% in media, and only 2% of Ag accumulated in aboveground parts.

It is well known that exposure of plants to HM stress affects the uptake and accumulation of essential nutrients [1,3,24,25]. This phenomenon is a result of several mechanisms, including competition for transporters or a direct impact of Ag ions on plasma membrane calcium channels [26]. Interestingly, 1 µM Ag evoked an increase in the Ca concentration in the shoots, while 10 µM Ag stimulated accumulation of Ca in the roots. The obtained results indicate a significant impact of Ag(I) ions on the Ca bioconcentration and translocation. Given the role of Ca in HM detoxification [26], the increasing content of Ca under Ag(I) exposure may indicate some role of this element in plant Ag resistance. The higher Ag(I) dose decreased the root K and P accumulation, and similar depletion of P has also been observed in *Spirodela polyrhiza* [16]. In contrast to our data, the exposure of *A. thaliana* to 2.0 mg L^−1^ of Ag(I) (ca. 18.5 μM) did not change the content of some mineral nutrients in the leaves; however, 2.0 mg L^−1^ of Ag nanoparticles significantly reduced the accumulation of K, Fe, and Zn in the leaves by 71%, 50%, and 49%, respectively [19].

Quantitative changes in LMWOAs are frequently observed in various species under HM stress due to their chelating ability. This can lead to reduction of HM phytoavailability (chelation of HMs with exudates) or their intercellular detoxification (chelation of HMs in the cytosol) [2,27,28]. Moreover, it has been pointed out that LMWOAs participate in long-distance translocation of HMs to parts of plants with low metabolic activity such as the cell wall or trichomes [27]. Here, we observed that the low concentration of Ag ions significantly increased the concentrations of malic and citric acids in both the shoots and roots. The phenomenon of elevated accumulation of LMWOAs under HM stress has been observed in higher plants [2]. In the present study, a dose-dependent impact of the Ag(I) treatments on the content of LMWOAs was observed. A similar effect was found in previous research, where a low Cd concentration increased the level of malic and citric acids in the shoots and roots of *C. acaulis*, but increased accumulation of these acids was detected only in the shoots at a high Cd dose (acute stress, data not published). The reduced content of LMWOAs in the roots of plants treated with 10 µM Ag may be a result of increased exudation limiting the metal availability in the medium. On the contrary, the low Ag dose stimulated the accumulation of LMWOAs in the organs, indicating intercellular metal chelation as a detoxification mechanism.

Phytochelatins are essential intercellular ligands of HMs, and their content significantly increased in response to both Ag(I) doses. Ag belongs to a group of metals that induce PC synthesis in plants and yeasts [29]. It was also shown that Ag is well bound by PCs [30]. However, we found low ability of Ag(I) to stimulate PC biosynthesis in the roots and no induction of PC synthesis in the shoots. The accumulation of these thiols in the roots of *C. acaulis* treated with Ag(I) was lower in comparison to their accumulation after Cd exposure (unpublished) or in other plant species stressed by Cd or Pb ([4] and references therein). This fact can be explained by the higher strength of Cd, Pb, or Zn ions in PC induction compared with Ag [31]. Moreover, it was noted that the PC content in *Ricinus communis* collected from an area of silver mines, contrary to Cd or Pb, was not correlated with the Ag(I) content in the plants [32]. Lack of PC induction by Ag ions was also observed earlier in *Scenedesmus vacuolatus* [33]. The level GSH (i.e., a precursor of PCs) increased in the shoots with the increasing Ag(I) concentration in the nutrient medium. At the same time, accumulation of GSH in the roots was suppressed by the higher Ag dose. In agreement with our data, an Ag dose of 20–60 mg L^−1^ (as AgNO_3_) strongly elevated the GSH content in wheat callus cultures after 24 h of exposure [34]. GSH together with AsA are part of the nonenzymatic scavenging system [35]. It has been shown that these compounds protect plant cells against HM-induced oxidative damage [36,37]. The elevated level of GSH (but not AsA) in the leaves under the Ag(I) excess may indicate a protective role of this compound in the leaf cells under Ag(I) exposure. In turn, the higher Ag(I) concentration significantly decreased both GSH and AsA accumulation in the roots, which was in agreement with earlier data [38]. This depletion of antioxidant systems may have been evoked by the higher Ag amounts in the root tissue with consequent reduction of growth and vitality.

Since they typically contain one or more free hydroxyl groups, phenolic compounds play a significant role in reactive oxygen species (ROS) scavenging [39]. We found that the Ag(I) stress stimulated the accumulation of both major phenolic acids (chlorogenic and 3,5-dicaffeoylquinic). This fact indicates that an increase in the level of phenolic compounds may participate in Ag resistance mechanisms. The chlorogenic acid level in chamomile tissues (Asteraceae family) has been observed to increase under various doses of different metals, which indicates a more general contribution to the antioxidative potential of plants [5]. Although there is evidence that HM ions can change the synthesis and accumulation of phenolic compounds in plants, the influence of HMs on this process is highly dependent on the level of the stress factor [2,15]. Khan et al. [40] showed that *Pennisetum glaucum* exposed to increasing concentrations of Ag (2, 4, and 6 mM) for 24 h exhibited approximately 2-fold higher total phenolic and flavonoid contents compared with control plants. Similarly, an increasing effect of Ag ions on the accumulation of phenolic compounds was observed in *Bacopa monnieri* [41] and *Hordeum vulgare* [42]. In turn, *Carlina vulgaris* collected from a highly HM-polluted area accumulated lower amounts of phenolic compounds and flavonoids than plants inhabiting nonpolluted areas [15].

The lower dose of Ag(I) enhanced accumulation of both triterpene acids (oleanolic and ursolic), while an elevated level of carlina oxide in the roots was found at the higher dose of Ag(I). The role of triterpenes in protection against HM ions has been considered previously, as these compounds have antioxidant properties and can protect plant cells against ROS damage [43]. Previous studies showed that HM stress elevated the ursolic acid level in *Prunella vulgaris* [44], while Cd stress increased accumulation of oleanolic acid in *Achrynthes bidentate* cell cultures [45].

## 4. Materials and Methods

### 4.1. Plant Materials and Growth Conditions

*C. acaulis* L. achenes collected from the Botanical Garden of Maria Curie-Skłodowska University in Lublin (voucher specimen no. 2005A) were germinated on the surface of garden soil. The young seedlings (10-days old) were transferred into polyethylene pots filled with garden soil. The plants (one per pot) were cultivated over 28 days to achieve an approximately 12 cm diameter of the leaf rosette. Afterwards, the plants were carefully washed with distilled water and transferred into pots filled with 0.5 L of half-strength Hoagland’s solution. The plants were acclimated to hydroponic conditions for 5 days and thereafter divided into three groups (25 plants per treatment): (i) control plants cultivated without addition of Ag, (ii) plants cultivated with 1 µM of Ag, and (iii) plants cultivated with 10 µM of Ag. Silver was added in the form of nitrate (AgNO_3_, Sigma-Aldrich, St. Louis, MO, USA). The plants were grown in controlled conditions in a growth chamber at 18/25 °C (night/day) under light-emitting diodes at a photosynthetic photon flux density of 150 µmol m^−2^ s^−1^ and relative humidity of 60%–65%. The plants were harvested 14 days after the addition of silver, separated into shoots and roots (the roots were washed with deionized water), and weighed to determine fresh biomass. For the determination of LMWOAs, PCs, GSH, and AsA, the samples (biological repeats n = 5) were frozen in liquid nitrogen and stored at −80 °C. Similar aliquots were dried at room temperature for determination of secondary metabolites and at 70 °C to constant weight for measurement of Ag and mineral nutrients (biological repeats n = 4).

### 4.2. Determination of Silver and Mineral Nutrients

Aliquots of powdered samples were digested in a 5 mL mixture of HNO_3_:H_2_O (2:8 *v*/*v*) in a microwave digestion apparatus (TOPwave, Analytick Jena AG, Jena, Germany). The content of elements was measured using an ICP-OES PlasmaQuant PQ 9000 Elite (Analityk Jena AG, Jena, Germany). The effective plasma power was 1300 W, and the argon flow rate of the plasma, auxiliary, and nebulizer were 12, 0.5, and 0.6 L/min, respectively.

### 4.3. Analysis of LMWOAs, Ascorbic Acid, and Thiols

An Agilent 7100 Capillary Electrophoresis (Agilent Technologies, Santa Clara, CA, USA) was applied for analysis of LMWOAs, AsA, GSH, and PCs according to protocols published previously [37,46,47].

### 4.4. Quantification of Secondary Metabolites

Triterpenes (ursolic and oleanolic acid), chlorogenic acid, 3,5-dicaffeoylquinic acid, and carlina oxide were analyzed in 100% methanolic extracts using high-performance liquid chromatography (VWR Hitachi Chromaster 600 chromatograph, Merck, Darmstadt, Germany). The triterpenes were analyzed using the RP18e LiChrosper 100 column (Merck, Darmstadt, Germany) (25 cm × 4.9 mm i.d., 5 µm particle size) according to the method reported previously [48]. The C18 reversed-phase column Kinetex (Phenomenex, Torrance, CA, USA) was used to separate phenolic acids as described in the previous work [49]. The carlina oxide content was measured according to the method reported previously [50].

### 4.5. Visualization of H_2_O_2_

Hydrogen peroxide (H_2_O_2_) in the *C. acaulis* leaves was visualized using the 3′,3-diaminobenzidine method [51].

### 4.6. Statistical Analysis and Experimental Design

The completely randomized design of the experiment involved three treatments (control, 1 µM Ag, and 10 µM Ag) with 25 plants per treatment. The whole experiment was performed two times in the same growth conditions. The data were statistically analyzed using one-way analysis of variance (ANOVA). The differences between the treatments were determined with Tukey’s test at the 0.05 probability level. Statistic ver. 13.3 software (TIBCO Software Inc. 2017, Palo Alto, CA, USA) was used to carry out all statistical analysis.

## 5. Conclusions

Our data confirm that the responses of *C. acaulis* to Ag(I) are dose dependent. Although Ag(I) slightly increased the H_2_O_2_ concentrations in the leaves, its accumulation in the shoots was low. On the contrary, Ag was mainly retained in the roots, but the low Ag(I) dose even stimulated root growth. Only the higher Ag(I) concentration disturbed the K, P, and Ca balance, while the low Ag(I) stress affected Ca and Cu accumulation. The stimulation of the content of LMWOAs in plants exposed to 1 µM Ag together with the significantly higher content of phenolic and triterpene acids observed in this treatment may indicate both chelation of Ag(I) and induction of antioxidative mechanisms against Ag excess. In contrast, the higher Ag dose generally reduced the content of organic (malic and citric) acids, AsA, or GSH in the roots and, in combination with the relatively high accumulation of Ag in the roots, may be the cause of growth depression.

## Figures and Tables

**Table 1:** Concentration of selected mineral nutrients in the shoot and roots of *C. acaulis* grown under two Ag(I) treatments for 14 days. Data are mean ± SE (n = 4); values in the columns followed by the same letter are not significantly different between the treatments (*p* < 0.05, Tukey’s test).

Treatment	K (mg g^−1^ DW)		P (mg g^−1^ DW)	
	Shoot	Root	Shoot	Root
Control	55.45 ± 3.83 a	41.93 ± 2.35 a	3.48 ± 0.35 a	5.69 ± 0.37 a
1 µM Ag	55.01 ± 4.52 a	40.17 ± 5.22 a	3.46 ± 0.65 a	5.79 ± 0.48 a
10 µM Ag	52.02 ± 1.53 a	25.19 ± 2.19 b	2.75 ± 0.23 a	3.84 ± 0.19 b
	**Ca (mg g^−1^ DW)**		**Mg (mg g^−1^ DW)**	
	Shoot	Root	Shoot	Root
Control	12.22 ± 0.73 b	5.09 ± 0.42 b	4.26 ± 0.31 a	2.32 ± 0.09 a
1 µM Ag	14.37 ± 0.47 a	5.49 ± 0.47 b	4.20 ± 0.56 a	2.74 ± 0.03 a
10 µM Ag	9.26 ± 0.21 c	7.67 ± 036 a	3.75 ± 0.21 a	2.52 ± 0.04 a
	**Zn (µg g^−1^ DW)**		**Cu (µg g^−1^ DW)**	
	Shoot	Root	Shoot	Root
Control	21.57 ± 4.02 a	58.76 ± 7.82 a	5.32 ± 0.17 a	14.06 ± 1.40 a
1 µM Ag	24.89 ± 2.90 a	61.48 ± 12.02 a	3.73 ± 0.41 b	11.54 ± 1.14 a
10 µM Ag	20.64 ± 2.86 a	56.08 ± 9.68 a	4.54 ± 0.11 ab	14.92 ± 2.17 a
